# Plasmonic photocatalyst-like fluorescent proteins for generating reactive oxygen species

**DOI:** 10.1186/s40580-018-0140-7

**Published:** 2018-03-22

**Authors:** Jung Woo Leem, Seong-Ryul Kim, Kwang-Ho Choi, Young L. Kim

**Affiliations:** 1 Weldon School of Biomedical Engineering, Purdue University, West Lafayette, IN 47907 USA; 2 Department of Agricultural Biology, National Institute of Agricultural Sciences, Rural Development Administration, Wanju, Jeollabuk-do 55365 Republic of Korea; 3 Regenstrief Center for Healthcare Engineering, West Lafayette, IN 47907 USA; 4 Purdue Quantum Center, Purdue University, West Lafayette, IN 47907 USA

**Keywords:** Plasmonic photocatalysis, Fluorescent proteins, Photosensitization, Reactive oxygen species, Visible light

## Abstract

The recent advances in photocatalysis have opened a variety of new possibilities for energy and biomedical applications. In particular, plasmonic photocatalysis using hybridization of semiconductor materials and metal nanoparticles has recently facilitated the rapid progress in enhancing photocatalytic efficiency under visible or solar light. One critical underlying aspect of photocatalysis is that it generates and releases reactive oxygen species (ROS) as intermediate or final products upon light excitation or activation. Although plasmonic photocatalysis overcomes the limitation of UV irradiation, synthesized metal/semiconductor nanomaterial photocatalysts often bring up biohazardous and environmental issues. In this respect, this review article is centered in identifying natural photosensitizing organic materials that can generate similar types of ROS as those of plasmonic photocatalysis. In particular, we propose the idea of plasmonic photocatalyst-like fluorescent proteins for ROS generation under visible light irradiation. We recapitulate fluorescent proteins that have Type I and Type II photosensitization properties in a comparable manner to plasmonic photocatalysis. Plasmonic photocatalysis and protein photosensitization have not yet been compared systemically in terms of ROS photogeneration under visible light, although the phototoxicity and cytotoxicity of some fluorescent proteins are well recognized. A comprehensive understanding of plasmonic photocatalyst-like fluorescent proteins and their potential advantages will lead us to explore new environmental, biomedical, and defense applications.

## Introduction

Photocatalysis has extensively been used in a variety of applications, including energy generation, environment remediation, and biomedicine, as mentioned in numerous review articles on photocatalysis [1–8]. Conventional photocatalysis requires three essential components of a semiconductor photocatalyst, a light source with appropriate wavelengths, and an oxidizing agent (e.g. water or oxygen molecules). In semiconductor photocatalysis, the wide bandgap energy (e.g. 3.0–3.2 eV) of semiconductor photocatalysts intrinsically limits light absorption to only the ultraviolet (UV) region (wavelength of light λ < 420 nm), which accounts for only about 4% of the total solar energy. Furthermore, the requirement of UV irradiation is commonly considered as a serious biohazard, potentially leading to premature aging of the skin, suppression of the immune system, damage to the eyes, and skin cancer [9–12]. Thus, to avoid the use of UV as an activation light source, plasmonic effects of metal nanoparticles (mNPs), such as Au, Ag, and Pt, have been successfully hybridized, resulting in broad and strong light absorption in the visible region [13–16], as summarized in several recent review articles [17–21].

One of the important aspects of photocatalysis is photoinduced production of reactive oxygen species (ROS), which often have direct applications for environment remediation and biomedicine, such as disinfection, water purification, and air purification. Typical semiconductor photocatalysts, such as titanium dioxide (TiO_2_) and zinc oxide (ZnO), were extensively studied for efficient and stable photogeneration of ROS [1–6, 22]. As intermediate or final products, semiconductor photocatalysis generates several different types of ROS, including superoxide anion (O_2_^•‒^), singlet oxygen (^1^O_2_), hydrogen peroxide (H_2_O_2_), and hydroxyl radical (–OH^•^). Regarding ROS produced by plasmonic photocatalysis, O_2_^•‒^ and ^1^O_2_ are typically generated via electron transfer under visible light excitation [13, 14]. Overall, O_2_^•‒^ and ^1^O_2_ play a key role in electrochemistry and photochemistry related to photocatalysis.

There is always an imperative need for cost-effective, eco-friendly, and nontoxic photocatalytic nanomaterials and their photoexcitation using visible (or solar) light. Although plasmonic photocatalysis overcomes the requirement of UV irradiation, it still has concerns with respect to environmental and biomedical utilizations. For example, nano-sized plasmonic photocatalysts (e.g. 1 − 100 nm) could potentially have hazardous and adverse (e.g. carcinogenic and cytotoxic) biological effects, which often result in the limited utilizations for environmental remediation and biomedicine [23, 24]. Noble metals (e.g. Ag, Au, and Pt) also have some drawbacks, including rarity, high cost, and easy dissolution (especially for Ag) upon exposure to air or humidity. In this respect, nontoxic organic photosensitizers (e.g. natural dyes or proteins) could potentially be an excellent alternative to noble mNP-based plasmonic photocatalysts, as photosensitization has a great similarity with visible light-driven plasmonic photocatalysis.

In this review article, we introduce plasmonic photocatalyst-like fluorescent proteins for ROS generation upon visible (or solar) light activation. Several recent review articles have extensively covered photosensitizing molecules found in nature (e.g. porphyrin and chlorophyll) [25–27] and genetically-encoded ROS-generating proteins for cellular functions and redox signaling pathways [28–30]. To the best of our knowledge, a systematic review on ROS photoproduction from fluorescent proteins has not yet been available, compared to plasmonic photocatalysis. First, we briefly describe the basic mechanisms of plasmonic photocatalysis and photosensitization in terms of ROS photogeneration. Second, we review selected photosensitizing proteins that can be compared with plasmonic photocatalytic nanomaterials in a parallel manner. Third, we discuss outlook based on the current state of understanding on ROS utilizations. An enhanced understanding of plasmonic photocatalysis and fluorescent protein photosensitization will allow us to take advantage of ROS generated from light-induced fluorescent proteins for unexplored environmental, biomedical, and defense applications.

## Basic mechanisms of plasmonic photocatalysis and photosensitization

### Visible light-driven plasmonic photocatalysis

**Fig. 1:**
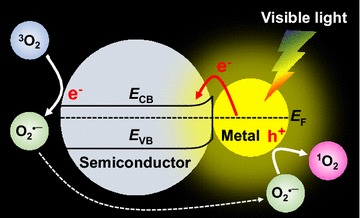
Schematic illustration of a plausible mechanism for generating O_2_^•‒^ and ^1^O_2_ (i.e. O_2_(^1^Δ_g_)) on metal–semiconductor hybrid nanostructures via hot electron transfer caused by surface plasmon resonance upon visible light excitation. *E*_CB_ and *E*_VB_ represent the conduction and valence bands of the semiconductor photocatalyst, respectively. *E*_F_ refers to the Fermi energy level

In general, plasmonic photocatalytic activities involve several different underlying mechanisms of electron and energy transfer depending on excitation energy and light sources, as summarized in the recent review articles [17–21]. The current consensus in the community is that visible light-driven plasmonic photocatalysis is mainly associated with generating two types of ROS (O_2_^•‒^ and ^1^O_2_) [13, 14]. Specifically, ROS generation from visible light-activated plasmonic photocatalysis can be summarized as follows (Fig. 1): In plasmonic photocatalysis using plasmon resonance excited by visible light, an electron transfer process from mNP to the semiconductor occurs at the metal/semiconductor interface. In general, Schottky barrier, which interrupts the electron transfer from mNP to the semiconductor, is formed at the junction interface between mNP and the semiconductor in the hybrid nanostructures due to the Fermi level difference between the two different materials. However, an electron can travel to the adjacent semiconductor if the plasmonic excitation energy is higher than Schottky barrier. Such a highly energetic electron is often referred to as a ‘hot’ electron. As the energetic electron in mNP migrates to the conduction band (*E*_CB_) of the semiconductor, this process reduces molecular oxygen O_2_(^3^Σ_g_^−^) of triplet ground state (i.e. ^3^O_2_) to generate O_2_^•‒^ at the semiconductor surface. In the meantime, mNP can hold the positive hole. The positive hole remained in mNP further oxidizes the previously produced O_2_^•‒^ to generate additional ROS of ^1^O_2_ (i.e. O_2_(^1^Δ_g_)) [13, 14]. As a result, plasmonic photocatalysis can generate and release O_2_^•‒^ and ^1^O_2_ under visible light irradiation.

### Type I and Type II reactions of photosensitization

**Fig. 2:**
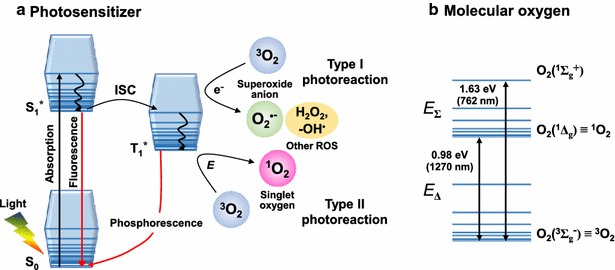
Jablonski diagram of photosensitizer and molecular oxygen (O_2_). **a** Photosensitization with the singlet ground (S_0_) and excited (S_1_^*^) states and their further interactions with O_2_. The triplet excited state (T_1_^*^) through an intersystem crossing (ISC) process can undergo electron (e^−^) transfer to the ground state of molecular oxygen O_2_(^3^Σ_g_^−^) (i.e. ^3^O_2_), generating superoxide anion (O_2_^•‒^) and other ROS products (e.g. H_2_O_2_ and –OH^•^) as Type I photoreaction. In addition, T_1_^*^ can undergo energy (*E*) transfer to ^3^O_2_, producing highly cytotoxic O_2_(^1^Δ_g_), commonly known as singlet oxygen (^1^O_2_), as Type II photoreaction. **b** Electronic configuration of the triplet ground state molecular oxygen O_2_(^3^Σ_g_^−^), its first (i.e. lowest-energy) singlet excited state O_2_(^1^Δ_g_), and its second (higher energy) singlet excited state O_2_(^1^Σ_g_^+^) [36, 37], where the superscripts 3 and 1 indicate triplet and singlet states, respectively. The energy gaps between the ground state and the two singlet excited states are shown in eV, including the corresponding luminescent wavelengths

Almost all photosensitizing molecules participate in Type I and/or Type II photoreactions involving the generation of ROS upon light activation (Fig. 2a) [31–34]. Predominant ROS generated by photosensitizers depends on a type of photosensitization reactions and a concentration of local electron acceptors. When light is incident on a photosensitizing molecule and light absorption occurs, the molecule is excited from the singlet ground state (S_0_) to the singlet excited state (S_1_^*^). The excited state loses the energy by returning back to S_0_ with fluorescent emission or through an intersystem crossing (ISC) process which involves conversion to the long-lived triplet excited state (T_1_^*^). T_1_^*^ can decay S_0_ via phosphorescent emission or can react with an electron donor molecule. In the latter case, O_2_^•‒^ is generated by electron transfer from the substrate in T_1_^*^ of the photosensitizer to ^3^O_2_ as Type I photoreaction. Because the most common electron acceptor is O_2_, O_2_^•‒^ can further interact with its surroundings to produce other reactive oxygenated products, such as H_2_O_2_ and -OH^•^. On the other hand, T_1_^*^ can also transfer the energy directly to ^3^O_2_, producing singlet oxygen of the first (i.e. lowest-energy) singlet excited state ^1^O_2_ (i.e. O_2_(^1^Δ_g_)) as Type II photoreaction (Fig. 2b). O_2_(^1^Δ_g_) has energy (*E*) of 0.98 eV (*E*_Δ_) and its second (higher energy) singlet excited state O_2_(^1^Σ_g_^+^) is 1.63 eV (*E*_Δ_ +* E*_Σ_) above the triplet ground state (i.e. ^3^O_2_) [35–37]. O_2_(^1^Σ_g_^+^) decays extremely fast (~ picoseconds) to the first excited state ^1^O_2_ especially in aqueous media by its electronic-to-vibrational energy-transfer process [36–38]. Thus, the generation of O_2_(^1^Σ_g_^+^) in biology is often neglected. Similarly to visible light-activated plasmonic photocatalysis, Type I and Type II photoreactions of photosensitization can generate and release both O_2_^•‒^ and ^1^O_2_ under visible light activation.

### ROS lifetime and migration distance in plasmonic photocatalysis and photosensitization

As explained above, both plasmonic photocatalysis and photosensitization under visible light activation can produce short-lived ROS, given that O_2_^•‒^ and ^1^O_2_ are highly unstable and reactive [39, 40]. ROS photogenerated from plasmonic photocatalysis and photosensitization is only effective in the vicinity to semiconductor photocatalyst nanomaterials or photosensitizing molecules. Typically, O_2_^•‒^ exhibits a lifetime of ~ 50 μs, depending on the local environments [41]. On the other hand, the typical lifetime of ^1^O_2_ is ~ 3.1–3.9 μs in H_2_O. The lifetime of ^1^O_2_ can be as long as 68 μs in deuterium oxide (D_2_O), because it is mainly determined by energy transfer to the vibrational energy levels of the surrounding molecules [38, 42]. Short-lived ROS from plasmonic photocatalysis and photosensitization allows the migration distance to be as long as ~ 320 and ~ 200 nm for O_2_^•‒^ and ^1^O_2_, respectively [41, 43]. Overall, the short lifetime and the relatively short migration (or damage) distance can be considered as a disadvantage requiring a high concentration for a prolonged effect or an advantage for a safeguard, given O_2_^•‒^ and ^1^O_2_ are extremely reactive and toxic.

## Identification of phototoxic fluorescent proteins from biological studies

The phototoxicity and cytotoxicity of some fluorescent proteins are well known in different scientific communities. In cellular imaging, several nontoxic variants of phototoxic fluorescent proteins were successfully developed for cellular labeling and imaging in vivo [44–46]. In a contrary manner, phototoxic fluorescent proteins have extensively been employed as a means of selectively damaging target molecules in a localized region and at a particular time-point upon light activation [47–49]. Chromophore photoreduction in red fluorescent proteins (RFPs) is considered to be mainly responsible for photobleaching and phototoxicity, forming dianionic open-shell states of the chromophore in RFPs [50]. This method is known as chromophore-assisted light inactivation (CALI) that can be used to inactivate target cells and ablate tissue of interest. In particular, CALI using fluorescent proteins can allow for spatiotemporal knockdown or loss-of-function of targeted proteins, which can be microscopically controlled with light activation in situ [30, 47, 49, 51]. In addition, some fluorescent proteins can be used for photodynamic therapy (PDT) to destruct diseased tissue without affecting the surrounding healthy tissue [52–54].

CALI and PDT using fluorescent proteins can offer an initial overview to identify major ROS-generating fluorescent proteins. There are several studies on CALI using photosensitizing proteins, such as enhanced green fluorescent protein (EGFP) [55, 56], mini Singlet Oxygen Generator (miniSOG) [57], KillerRed [58], and SuperNova [59]. CALI with EGFP was used to inactivate α-actinin in fibroblasts, which resulted in stress fiber detachment [55]. EGFP variants, including enhanced yellow fluorescent protein (EYFP) and enhanced cyan fluorescent protein (ECFP), were used for CALI. In general, the efficiency followed an order of EGFP > EYFP > ECFP [56]. The use of miniSOG for CALI was demonstrated [57], in which miniSOG was fused with the succinate dehydrogenase complex subunit of the mitochondrial respiratory complex II to disrupt complex II activity. Mitochondrion-targeted miniSOG caused rapid and effective death of neurons in a cell-autonomous manner without detectable damages to the surrounding cells [52]. Immunophotosensitizer 4D5 single chain variable fragment (4D5scFv)-miniSOG was used to selectively recognize the extracellular domain of human epidermal growth factor receptor 2 (HER2/neu) [53]. KillerRed was used for CALI of *Escherichia coli* and eukaryotic cells [58, 60, 61]. KillerRed was also tested for PDT by fusing to an antibody to target tumor cells, resulting in tumor-specific cell death [54]. SuperNova, which is a monomeric variant of KillerRed, was used to suppress actin filament motility by illuminating orange light [59].

## ROS photogeneration of phototoxic fluorescent proteins

The main underlying mechanism by which the aforementioned fluorescent proteins are phototoxic and cytotoxic is that these proteins are capable of generating and releasing several different types of ROS. The optical absorption and emission of phototoxic fluorescent proteins and their detected ROS types are summarized in Table 1. To the best of our knowledge, this table provides a comprehensive list of phototoxic fluorescent proteins that can generate and release ROS upon visible light excitation and activation in a comparable manner of visible light-activated plasmonic photocatalysis.

**Table 1:** Optical excitation and emission of phototoxic fluorescent proteins and their detected ROS types

Fluorescent protein variant	Excitation maximum (nm)	Emission maximum (nm)	Detected ROS type	References
GFP	395/476	503/509	^1^O_2_	[62]
EGFP	488	507	^1^O_2_	[63]
miniSOG	448	500	^1^O_2_	[64, 65]
SOPP	439	488/515	^1^O_2_	[66]
Pp2FbFP L30 M	449	495	^1^O_2_	[67]
KillerRed	585	610	O_2_^•‒^ and ^1^O_2_	[59, 68, 69]
SuperNova	579	610	O_2_^•‒^ and ^1^O_2_	[59]
TagRFP	555	584	^1^O_2_	[70]
mKate2	588	633	O_2_^•‒^ and ^1^O_2_	[71]

### GFP and EGFP

**Fig. 3:**
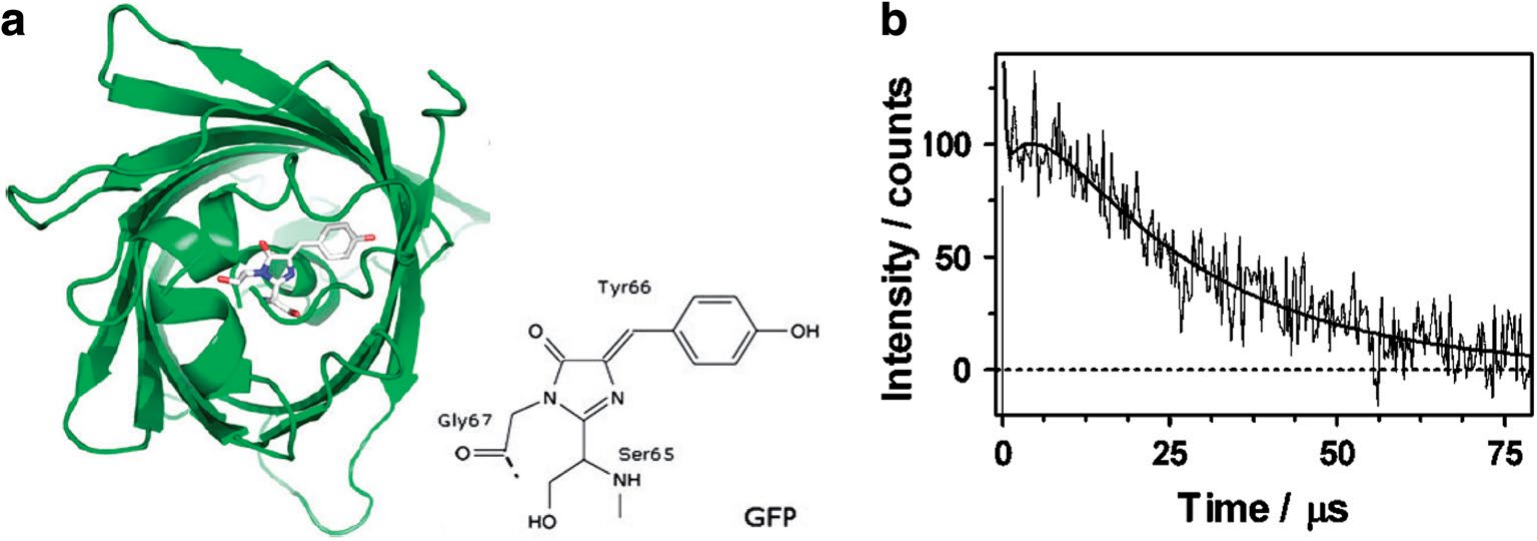
**a** 3D structure of GFP (PDB ID: 1GFL) obtained from X-ray crystallography and its mutant Ser65Thr (PDB ID: 1EMA). GFP has a *β*-barrel structure with the chromophore embedded in its core (Reproduced from [79] with the permission of Royal Society of Chemistry). The chromophore is shown in its neutral form with protonated phenolic oxygen. **b** Normalized time-resolved phosphorescent intensity. Emission signals of ^1^O_2_ generated from EGFP irradiated at λ_ex_ = 532 nm in deuterated phosphate-buffered saline (d-PBS) (1:3) were detected at λ = 1275 nm (Reproduced from [63] with the permission of Elsevier)

GFP was first discovered by Shimomura et al. [72] as a companion protein to the famous chemiluminescent protein (i.e. aequorin) from *Aequorea* jellyfish. Since then, GFP has revolutionized cell biology and cellular imaging [73]. As an electron donor, GFP was also utilized for converting light-to-electricity in photodetectors or photovoltaic cells [74–77]. GFP has a unique cylindrical (can)-like shape consisting of an 11-strand *β*-barrel with a single α-helical strand containing a chromophore. The GFP chromophore is *p*-hydroxybenzylidene-imidazolinone formed from residues 65–67 and is almost perfectly buried in the center of *β*-can (Fig. 3a) [78, 79]. As far as ROS is concerned, GFP and EGFP are typically known to produce ^1^O_2_ via Type II photoreaction under excitation at blue light of λ = 400–500 nm [62, 63]. In particular, ^1^O_2_ production ability of GFP is considered to attribute to the accessibility of molecular oxygen to the chromophore [80]. ^1^O_2_ was detected in GFP-expressing *Escherichia coli* bacteria and kidney cells by means of electron spin resonance (ESR); singlet oxygen spin-trap 2,2,6,6-tetramethyl-4-piperidinyloxy (TEMP) was bound to ^1^O_2_ to produce a stable secondary radical 2,2,6,6-tetramethylpiperidine-1-oxyl (TEMPO) and the TEMPO quantities were measured to correlate with the concentration of ^1^O_2_ by measuring ESR spectra [62]. ^1^O_2_ produced by EGFP in a solution was also measured by time-resolved near-infrared luminescence measurements at λ = 1275 nm (Fig. 3b) [63].

### miniSOG

**Fig. 4:**
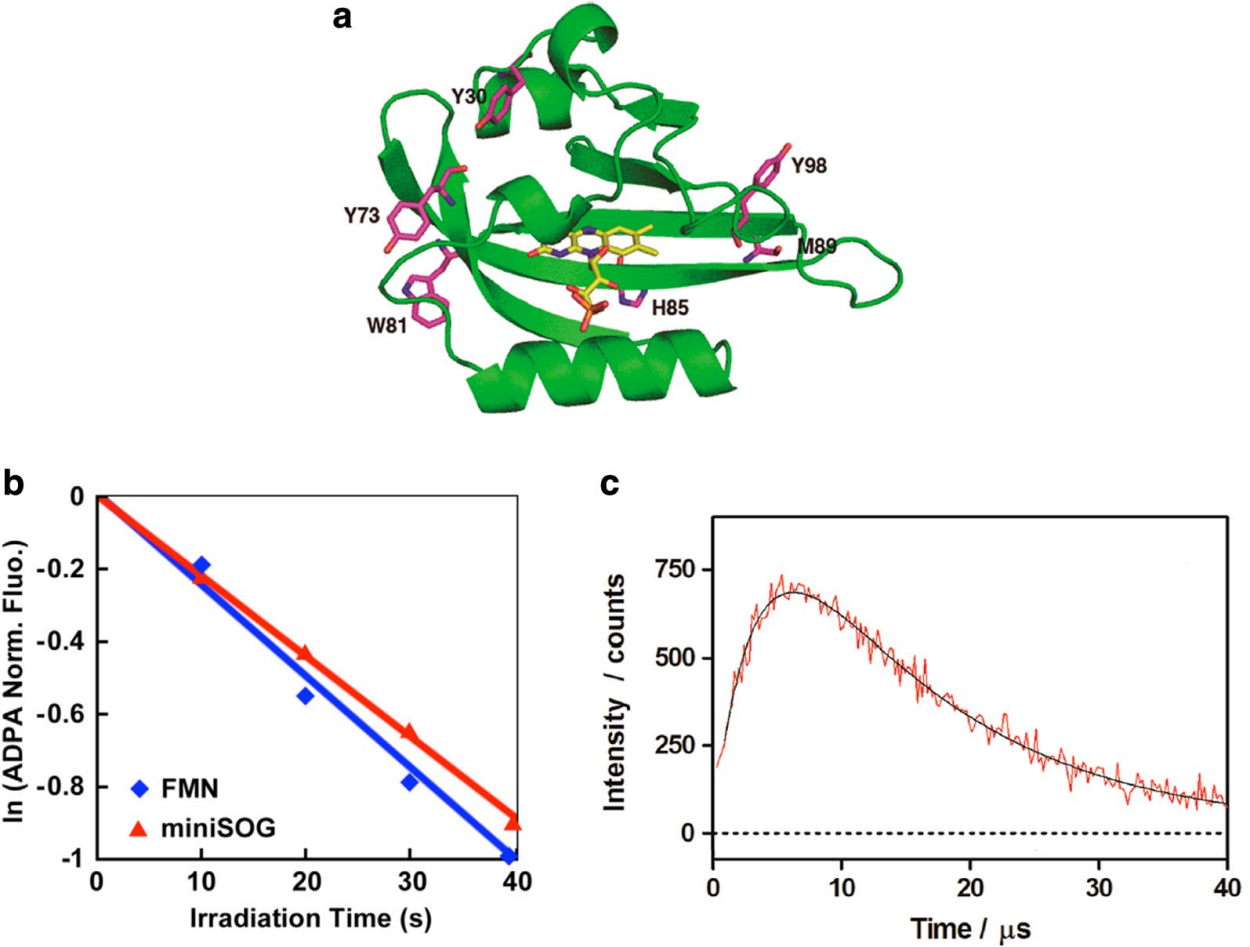
**a** 3D structure of miniSOG. This molecular model is based on the structure of the improved LOV protein (PBD ID: 4eet) using the Swiss-model server [83]. The backbone of miniSOG is shown as the green ribbon, flavin mononucleotide (FMN) as the orange sticks, and the amino acids as the magenta sticks (Reproduced from [65] with the permission of American Chemical Society). **b** Degradation of ADPA reacted with ^1^O_2_ photogenerated by miniSOG under light irradiation (red) (Figure from [64] and Creative Commons license). **c** Photosensitized ^1^O_2_ formation from miniSOG. Time-resolved ^1^O_2_ phosphorescent signals at λ = 1275 nm were recorded in d-PBS upon pulsed laser excitation at λ_ex_ = 355 nm (Reproduced from [65] with the permission of American Chemical Society)

By engineering the light-oxygen-voltage (LOV) domain of *Arabidopsis thaliana* phototropin 2 (*AtPhot2*), fluorescent flavoprotein miniSOG was originally developed to improve correlative light and electron microscopy [64, 65]. In terms of sizes (number of amino acids), miniSOG contains 106 amino acids, which is less than half the size of GFP (Fig. 4a). miniSOG is excited maximally at λ_ex_ = 448 nm and emits green light with two peaks at λ_em_ = 500 and 528 nm [64]. Regarding ^1^O_2_ photogenerated from miniSOG, ^1^O_2_ was detected using anthracene-9,10-dipropionic acid (ADPA) as a turn-off sensor probe of ^1^O_2_ [64] and ^1^O_2_ phosphorescent signals (Fig. 4b, c) [65]. After ADPA reacted with ^1^O_2_, it was converted to an endoperoxide form, which led to a decrease in fluorescence at λ_em_ = 406 nm [81]. ^1^O_2_ photogeneration of miniSOG is also supported the idea that the chromophore is accessible to oxygen molecules [82]. In addition, as an improved mutant of miniSOG, singlet oxygen photosensitizing protein (SOPP) was developed to achieve more efficient photogeneration of ^1^O_2_ [66].

### Pp2FbFP L30 M

**Fig. 5:**
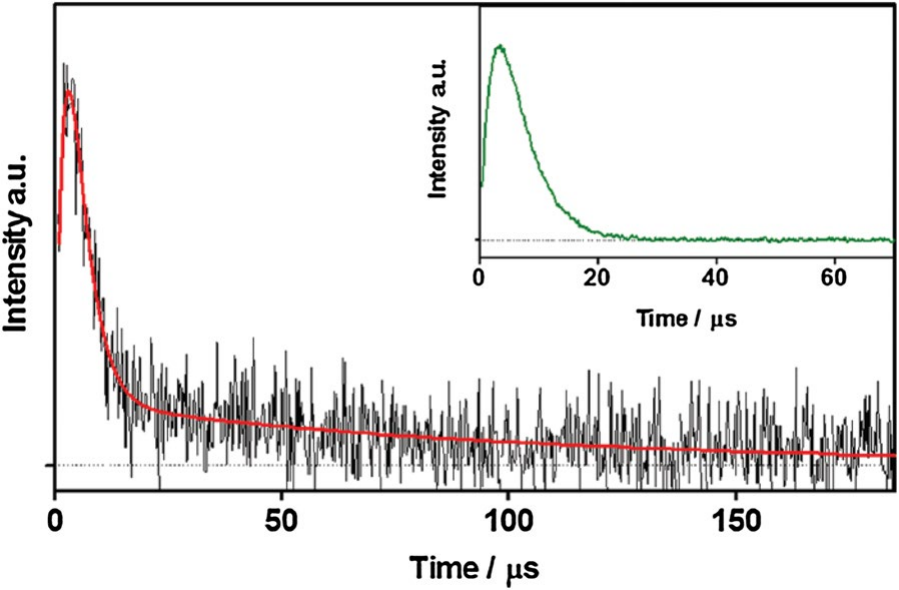
Time-resolved ^1^O_2_ phosphorescent signals for Pp2FbFP L30 M (λ_ex_ = 355 nm) in an air-saturated PBS solution at λ = 1275 nm. The corresponding trace for FMN was included in the inset for comparison to show the absence of the long-lived tail (Reproduced from [67] with the permission of Royal Society of Chemistry)

Pp2FbFP L30 M was derived from *Pseudomonas putida* flavin-binding Pp2FbFP with a further mutation of L30 M, which was originated from blue-light photoreceptors of the LOV family [67]. Upon light excitation, ^1^O_2_ photoproduction of Pp2FbFP L30 M was detected by measuring phosphorescent emission of ^1^O_2_ at λ_em_ = 1275 nm (Fig. 5) [67].

### KillerRed

**Fig. 6:**
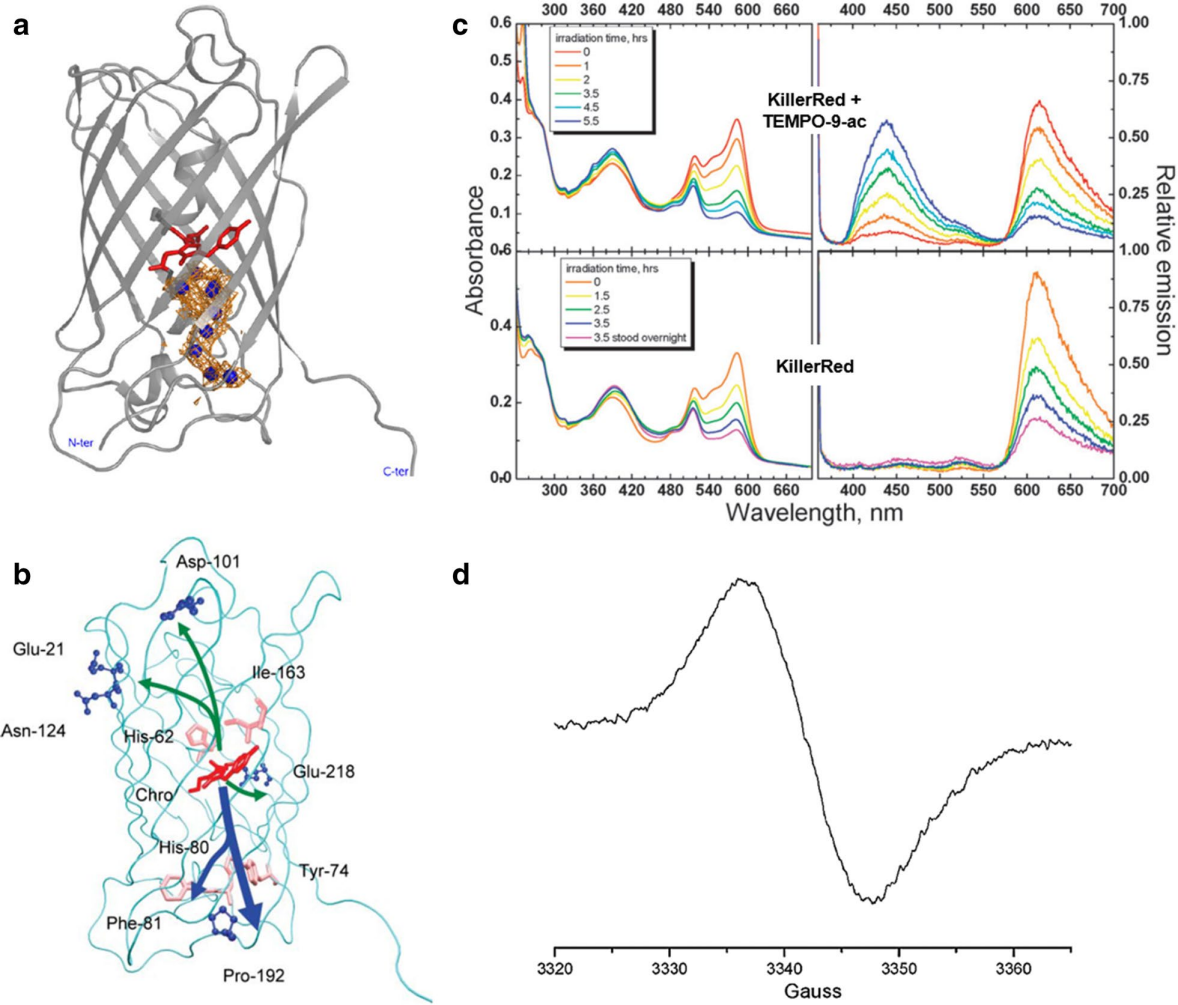
**a** 3D structure of a monomer of KillerRed obtained from X-ray crystallography (Reproduced from [85] with the permission of by John Wiley and Sons Inc.). Monomer A is shown with the backbone represented in gray and the chromophore in red. The cavity forming the channel is shown as the orange isomesh at 1 Å above the van der Walls radius and water molecules in the channel are depicted as the blue spheres. The cleft-like opening channel filled with water (oxygen) molecules is located in the *β*-barrel frame between *β*7 and *β*10 sheets. **b** Possible escape routes for molecular oxygen in the simulations (Reproduced from [86] with the permission of Royal Society of Chemistry). The chromophore is shown in red, the residues at the exits in blue, and other important residues along the pathways in pink. The escape pathways are highlighted by arrows (major channels in blue and minor channels in green). **c**, **d** Detection of O_2_^•‒^ photogenerated from KillerRed (Reproduced from [68] with the permission of Royal Society of Chemistry). **c** Light absorption (left panels) and emission (right panels) of KillerRed with and without the radical fluorescent probe TEMPO-9-ac (λ_ex_ = 358 nm and λ_em_ = 440 nm). **d** Representative EPR spectrum of KillerRed with DMPO in a PBS solution under light irradiation at λ_ex_ = 560 nm, supporting O_2_^•‒^ generation

KillerRed is one of the most studied RFPs for phototoxicity and ROS photogeneration. Regarding its origin, KillerRed was derived from the jellyfish chromoprotein anm2CP [84]. KillerRed is composed of 11 anti-parallel *β*-sheets that form a barrel structure with a central chromophore of Q65-Y66-G67 [85, 86]. Owing to its efficient ROS photogeneration, KillerRed was reported to be strongly phototoxic upon light illumination in a wavelength range of λ_ex_ = 540–580 nm [50, 68], exceeding other fluorescent proteins at least 1000-fold [84]. The extremely high phototoxicity is considered to be mainly attributed from a unique (cleft-like) structural feature in the *β*-barrel frame between *β*7 and *β*10 sheets. This cleft-like structure has an opening channel filled with water (oxygen) molecules connecting the chromophore’s cavity with the exterior of the protein barrel [84–86] (Fig. 6a). The opening channel leading to the chromophore is considered to facilitate water and/or oxygen diffusion to/from the chromophore. Computational simulations also support the idea that the water channel can increase the chromophore’s accessibility to molecular oxygen (Fig. 6b) [86].

As far as ROS photogeneration is concerned, KillerRed is known to undergo Type I photosensitization reaction to yield O_2_^•‒^ [68]. Irradiated KillerRed exhibited a tenfold increase in fluorescence signals (λ_em_ = 440 nm) of 4-((9-acridinecarbonyl)amino)-2,2,6,6-tetramethylpiperidin-1-oxyl (TEMPO-9-ac), compared to unchanged levels of controls (Fig. 6c) [68]. As a turn-on fluorescent free radical probe for sensing ROS related Type I photosensitization, the original status of TEMPO-9-ac is not fluorescent as the acridine moiety is initially quenched by the stable paramagnetic nitroxide moiety. ROS (mostly long-lived carbon- or sulfur-centered) can convert nitroxide to the corresponding piperidine, resulting in fluorescence turn-on (λ_ex_ = 358 nm and λ_em_ = 440 nm) [68, 87, 88]. Irradiated KillerRed with 5,5-dimethyl-1-pyrroline-N-oxide (DMPO) in PBS showed a broad singlet with a peak-to-trough width of 15 Gauss in the electron paramagnetic resonance (EPR) spectrum, supporting O_2_^•‒^ generation. (Fig. 6d). As controls, non-irradiated KillerRed and irradiated PBS did not produce EPR signals [68]. ROS associated with Type I photosensitization reaction was also detected with spin trapping of DMPO using steady-state EPR [89, 90]. In addition to O_2_^•‒^, ^1^O_2_ was detected in irradiated KillerRed using a radical scavenger (sodium azide, NaN_3_) and a fluorescent probe (ADPA) [59, 69]. Thus, ROS photoinduced by KillerRed is primarily associated  to O_2_^•‒^ with a possibility of ^1^O_2_ photogeneration.

### SuperNova

**Fig. 7:**
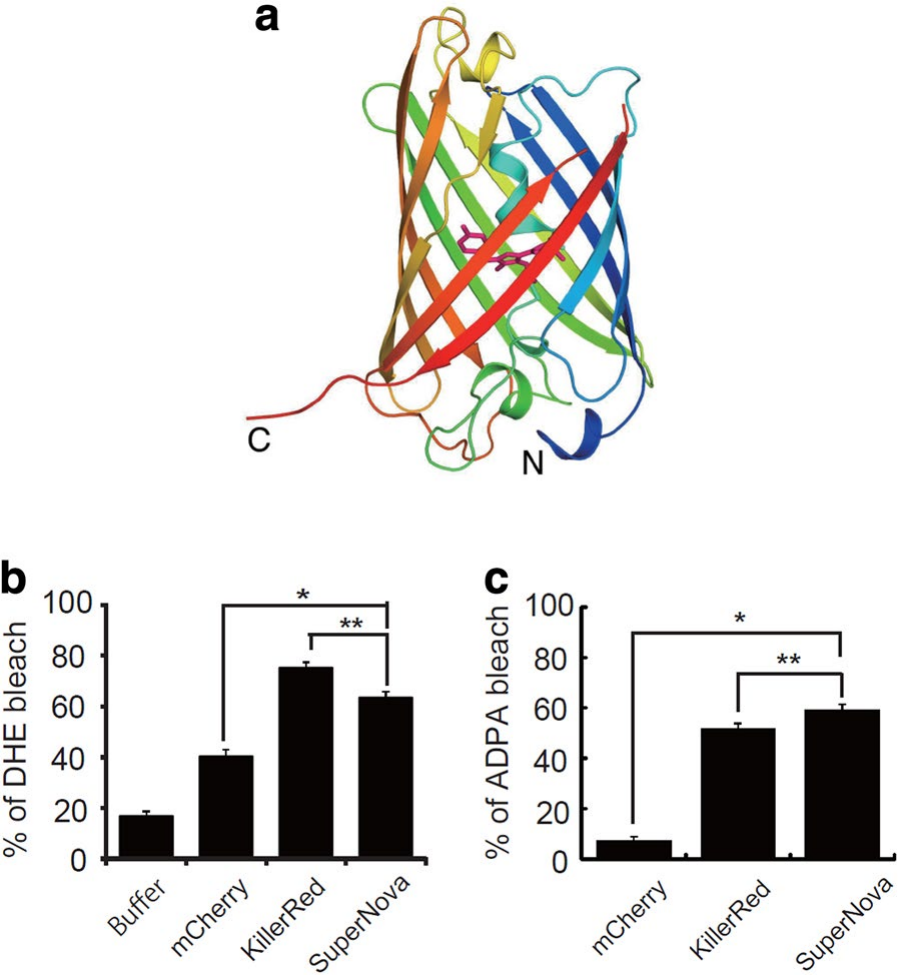
**a** 3D crystal structure of SuperNova (Reproduced from [59] with the permission of Springer Nature). SuperNova (monomer) is represented by the rainbow ribbon diagram and the chromophore is represented by the magenta stick model. **b**, **c** ROS detection generated from SuperNova (Reproduced from [59] with the permission of Springer Nature). **b** Detection of O_2_^•‒^ photogeneration of mCherry, KillerRed, and SuperNova by measuring bleaching of DHE fluorescence, including references (buffers). The irradiation condition was 0.73 W cm^−2^ for 10 min. **c** Detection of ^1^O_2_ photogeneration in mCherry, KillerRed, and SuperNova by measuring bleaching of ADPA fluorescence. The irradiation condition was 1.4 W cm^−2^ for 5 min. mCherry (λ_ex_ = 587 nm and λ_em_ = 610 nm) is a monomeric fluorescent protein variant derived from the *Discosoma* red (DsRed) protein [93–95]

SuperNova is a monomeric mutant of KillerRed. When KillerRed is fused to a protein of interest, it usually disrupts function and localization of other proteins due to its larger size and functional dimerization. To overcome this limitation, a monomeric variant was derived from KillerRed via random mutagenesis [59]. Specifically, SuperNova was developed (Fig. 7a), following six mutations compared with KillerRed: G3V, N145S, L160T, F162T, L172 K, M204T [59]. As a result, SuperNova is considered to have the similar photochemical properties as the parental protein (i.e. KillerRed), showing the excitation and emission maxima at λ_ex_ = 579 nm and λ_em_ = 610 nm, respectively. Both O_2_^•‒^ and ^1^O_2_ generated by SuperNova under orange light irradiation were detected using fluorescent probes of dihydroethidium (DHE) and ADPA, respectively [59]. In particular, enhanced photobleaching in DHE and ADPA supported photogeneration of O_2_^•‒^ and ^1^O_2_ from SuperNova, respectively (Fig. 7b, c) [59]. The original state of DHE exhibits blue fluorescent emission (λ_ex_ = 365 nm and λ_em_ = 435 nm) until being oxidized primarily by O_2_^•‒^. Oxidation of DHE results in hydroxylation at 2-position forming 2-hydroxyethidium, showing reduced blue fluorescent emission (i.e. bleaching) and increased red fluorescent emission (λ_ex_ = 490 nm and λ_em_ = 590 nm) [91, 92].

### TagRFP

**Fig. 8:**
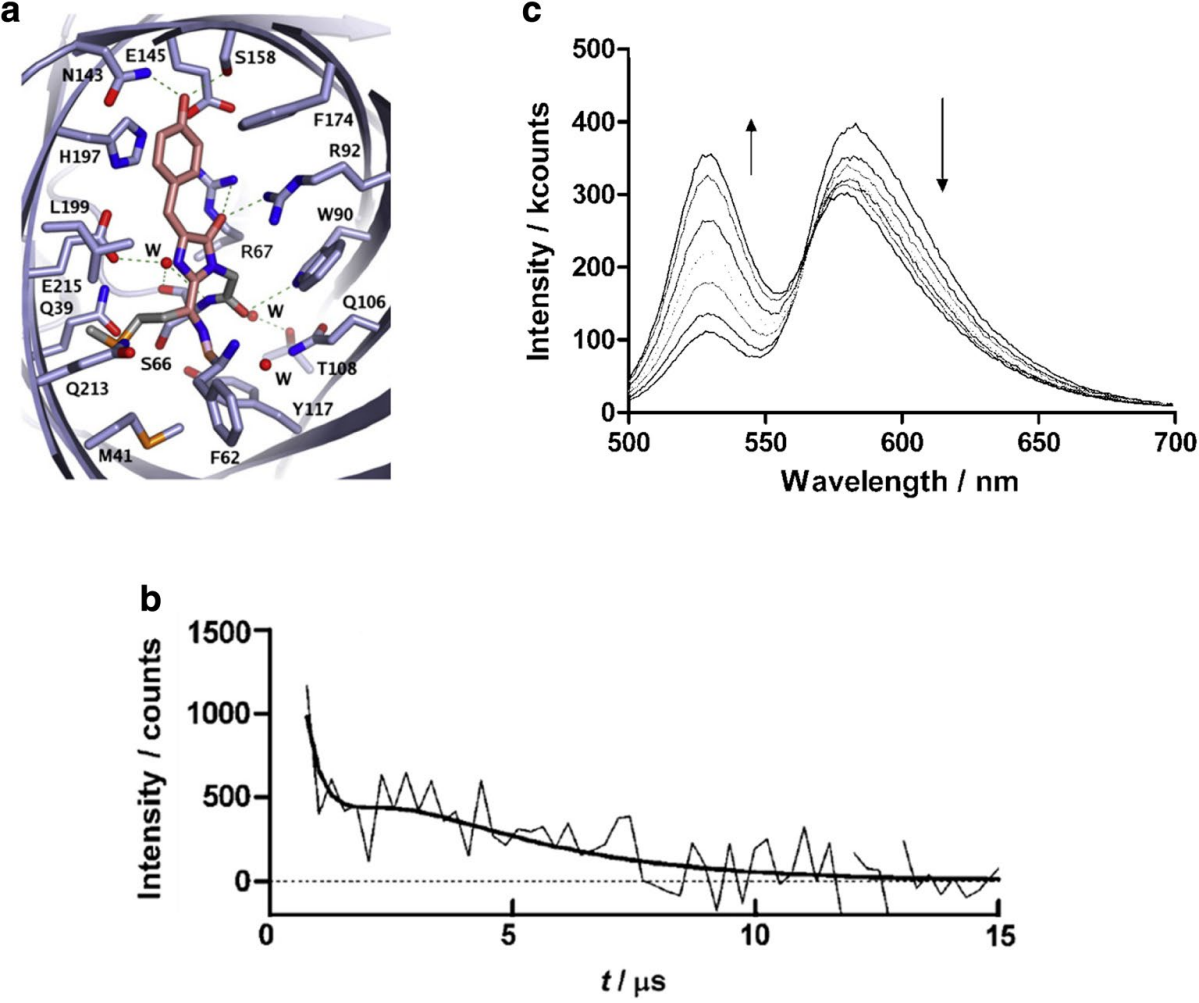
**a** Chromophore and their environment in TagRFP (Reproduced from [97] with the permission of Elsevier). The chromophore backbone for TagRFP is shown in orange. The hydrogen bonds are indicated with the green dashed lines, the atoms are colored by the atom type, and the water molecules are shown as the red spheres. **b**, **c** Detection of ^1^O_2_ photogenerated from TagRFP (Reproduced from [70] with the permission of John Wiley and Sons). **b** ^1^O_2_ phosphorescence photosensitized by TagRFP in a PBS:Glycerol:d-PBS mixture (1:1:20) upon irradiation at λ_ex_ = 532 nm. The luminescence signal at λ = 1270 nm was fit with a triexponential function. **c** Time course of fluorescent spectra of optically matched solutions of SOSG (λ_ex_ = 480 nm) and TagRFP under light irradiation at λ_ex_ = 532 nm. The increased amount of ^1^O_2_ was detected by the increase in the SOSG band (λ_em_ = 527 nm) with irradiation at λ_ex_ = 532 nm. The concomitant decrease in the TagRFP band (λ_em_ = 590 nm) indicated photobleaching and/or photoconversion

As a monomeric RFP, TagRFP was derived from the sea anemone *Entacmaea quadricolor* fluorescent protein TurboRFP (random mutant of eqFP578) (Fig. 8a) [96]. Unlike KillerRed [84, 85] and photosensitizing GFP mutants [80], TagRFP is known not to have a clear channel that connects the chromophore with the outside environment [97]. Thus, an alternative mechanism for oxygen diffusion in TagRFP was suggested such that transient protein permeability can play a role due to dynamical breathing [70]. This mechanism was also supported by the recent molecular dynamics simulations [86], in which the static picture offered by crystallography was explained by monitoring the triplet state. Regarding ROS photogeneration, TagRFP under green light (λ_ex_ = 532 nm) produced ^1^O_2_, which was confirmed by both time-resolved phosphorescence of ^1^O_2_ and a turn-on fluorescent probe of singlet-oxygen sensor green (SOSG) (Fig. 8b, c), even though O_2_^•‒^ was not detected using a fluorescent probe of DHE [70]. Specifically, pulsed laser irradiation (λ_ex_ = 532 nm) of TagRFP in an air-saturated mixture of PBS, glycerol, and d-PBS (1:1:20) allowed the detection of ^1^O_2_ phosphorescence at λ = 1270 nm (Fig. 8b), in which a fast spike in the earlier part of the signal (due to the scattered laser light and the sensitizer fluorescent emission) was followed by a slower rise and decay, corresponding to ^1^O_2_ kinetics. With the longer irradiation time, the enhanced SOSG fluorescence signals (λ_ex_ = 480 nm and λ_em_ = 527 nm) were also detected (Fig. 8c). Green fluorescent emission of SOSG corresponds to endoperoxide generated by an interaction of ^1^O_2_ with the anthracene component of SOSG [98, 99].

### mKate2

**Fig. 9:**
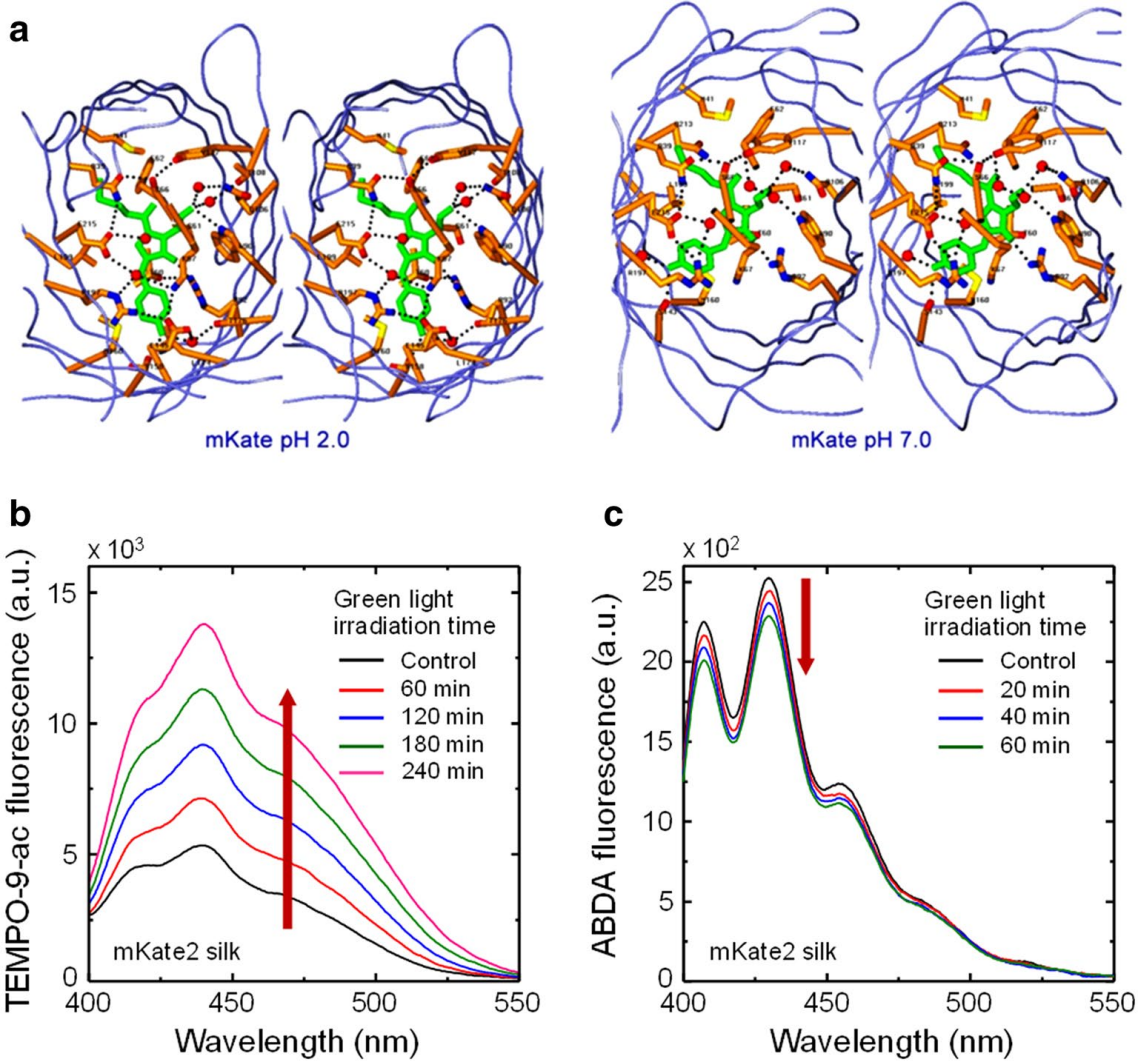
**a** 3D structure of the H-bond network in the vicinity of the trans chromophore (shown in green) for the mKate_pH2.0 structure (left) and the cis chromophore in the mKate_pH7.0 structure (right). Mediating waters are shown by red spheres (Reproduced from [102] with the permission of American Society for Biochemistry and Molecular Biology). **b**, **c** Detection of ROS generated by mKate2 embedded in silk (i.e. RFP fluorescent silk produced from silkworm transgenesis) using turn-on/off fluorescent probes upon green light irradiation at λ_ex_ = 532 nm [71]. Fluorescent emission signals of the probes were monitored from solutions containing mKate2 fluorescent silk. **b** O_2_^•‒^ mediated by Type I photosensitization reaction, captured by turn-on fluorescent signals of TEMPO-9-ac (λ_ex_ = 365 nm). **c** ^1^O_2_ mediated by Type II photosensitization reaction, detected by reduction in the original ABDA fluorescent intensity (λ_ex_ = 365 nm)

mKate2 is a far-red monomeric fluorescent protein with the maximal excitation wavelength (λ_ex_ = 588 nm) and emission wavelength (λ_em_ = 633 nm), which was derived from mKate containing three mutations of S165A, V48A, and K238R [100]. The mutation of S165A increases the fluorescent brightness, while the mutations of V48A and K238R accelerate protein maturation, including enhanced pH stability and photostability. Both mKate and mKate2 are widely considered as one of the phototoxic fluorescent proteins [44, 101], because of a cleft-like opening channel filled with water (oxygen) molecules in the *β*-barrel frame between *β*7 and *β*10 (Fig. 9a) [102]. ROS generated from mKate2 embedded in silk (i.e. RFP fluorescent silk produced from silkworm transgenesis [71, 103]) under green light irradiation (λ = 532 nm) was detected using fluorescent probes of TEMPO-9-ac and 9,10-anthracenediyl-bis(methylene)dimalonic acid (ABDA) for O_2_^•‒^ and ^1^O_2_, respectively (Fig. 9b, c) [71]. As the irradiation time of green light increased, the intensity of TEMPO-9-ac fluorescent peaks gradually enhanced and the ABDA fluorescent intensity decreased, compared to the baseline signals before light irradiation (controls). These result support the photogeneration of O_2_^•‒^ and ^1^O_2_ from mKate2. Similarly, ABDA was also widely used to detect the formation of ^1^O_2_ in solutions [71, 104, 105]; the original state of ABDA emits fluorescence at λ_em_ = 431 nm under photoexcitation at λ_ex_ = 380 nm and the oxidation of ABDA by ^1^O_2_ creates an endoperoxide, resulting in reduced fluorescent intensity [104].

### Fluorescent proteins with a cleft-like structure in *β*-barrels

**Fig. 10:**
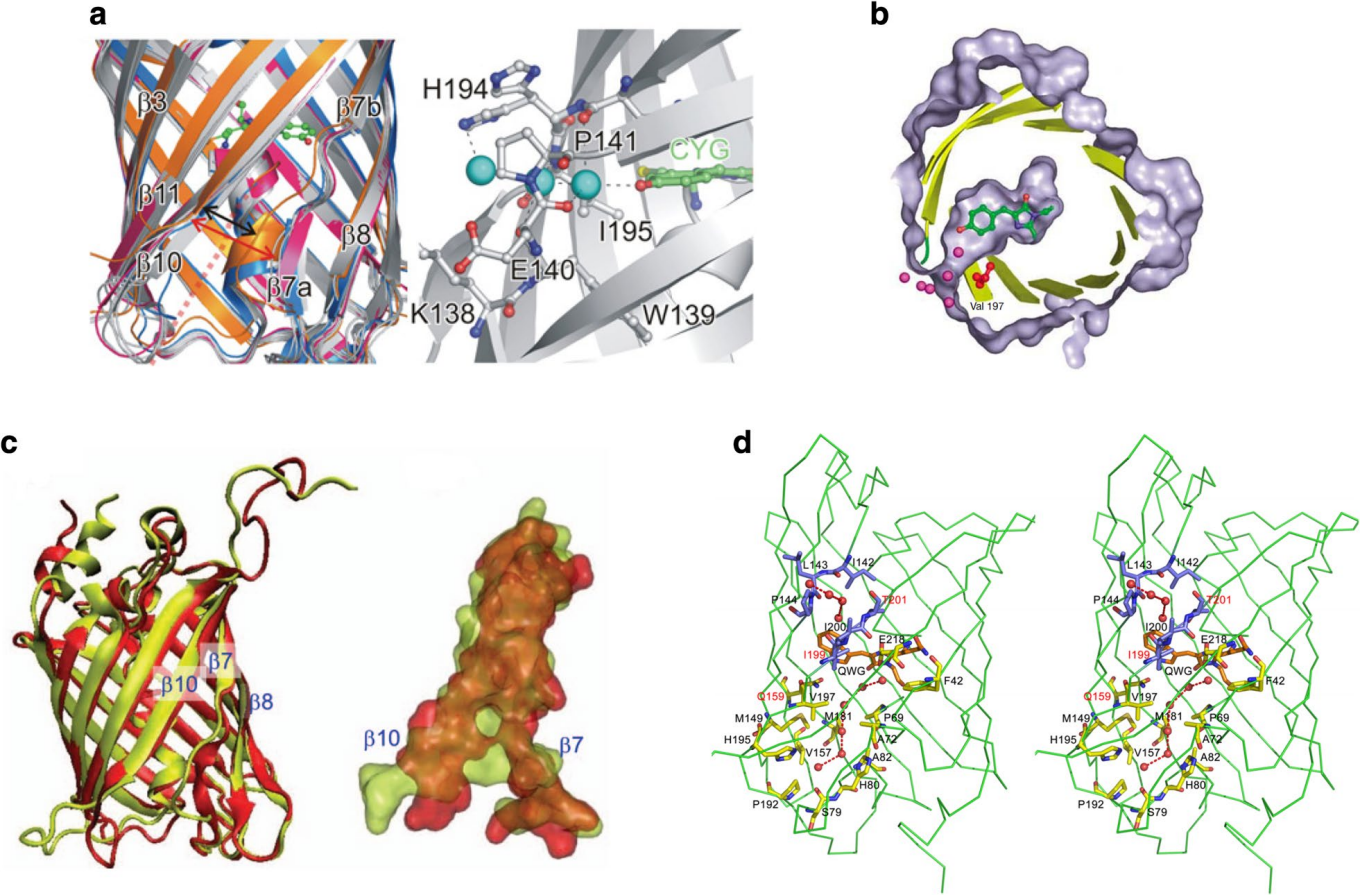
Cleft-like channels filled with water (oxygen) molecules in the *β*-barrel frame between *β*7 and *β*10 sheets of DronPa (**a**) (Reproduced from [106] with the permission of Portland Press Ltd.), TurboGFP (**b**) (Reproduced from [107] with the permission of John Wiley and Sons), mCherry (**c**) (Reproduced from [95] with the permission of AIP Publishing LLC.), and KillerOrange/mKillerOrange (**d**) (Figure from [108] and Creative Commons license). **a** DronPa: The cleft (indicated by the broken line) near to strand *β*7 is conserved. The red double headed arrow indicates the width of the cleft. Water molecules are shown as the cyan spheres, building hydrogen-bonded connection between the chromophore and the aqueous environment. The protein region around the opening and the chromophore is shown as the ball-and-stick model. **b** TurboGFP: A pore leading to the TurboGFP chromophore. The chromophore is highlighted in green and Val 197 in red. The protein surface (gray) is cut to show the pore and the chromophore cavity. The sections of secondary structure elements are shown as the yellow cartoons. Relevant water molecules are depicted as the magenta spheres. **c** mCherry: The superposition of ribbon structures of red (mCherry) and yellow (citrine) fluorescent proteins. The *β*7–*β*10 region is displayed with a space filling model to show that the gap in mCherry is larger than that in citrine. **d** KillerOrange (dimer) and mKillerOrange (monomer): The pore is filled by four water molecules connecting the indole moiety of the chromophore with the protein exterior. The water channel (residues shown in yellow) with a chain of seven water molecules (red spheres) and the pore (residues shown in blue) filled with four water molecules. The residues mutated in this work are labeled in red

Besides the aforementioned fluorescent proteins including KillerRed [85, 86] and mKate/mKate2 [71, 102], other fluorescent proteins could potentially be efficient in generating and releasing ROS upon visible light irradiation. As excellent candidates, several fluorescent proteins are known to contain a cleft-like water-filled channel in the *β*-barrel frame. Excellent examples include DronPa [106], TurboGFP [107], mCherry [95], KillerOrange/mKillerOrange [108], and zGFP506, zYFP538, zRFP574 [109] (Fig. 10). A water channel inside these fluorescent protein is suggested to open up solvent access to methylene/imidazolinone moieties of chromophores, allowing for enhanced generation and release of ROS, which in turn results in photobleaching and phototoxicity [44, 85, 110]. The water channel may also facilitate oxygen transport to a premature chromophore. This may promote the dehydrogenation step of chromophore maturation and transport abstracted proton transport outside the *β*-barrel, speeding up the chromophore’s maturation [107]. Indeed, ROS release from the fluorescent protein’s *β*-barrel through the water-filled channel may explain some phototoxicity and adverse effects (e.g. inhibition of cell division) of other fluorescent proteins in cellular imaging. Overall, these phototoxic fluorescent proteins warrant further detailed detection studies on photoinduced ROS and their exact types.

## Outlook and conclusion

We have discussed the similarities between plasmonic photocatalysis and phototoxic fluorescent proteins in terms of ROS generation under visible light activation. Like plasmonic photocatalysis, protein photosensitization requires three essential components of a fluorescent protein, a light source with appropriate wavelengths, and an oxidizing agent. A proper interaction of these elements leads to the photogeneration of ROS in the close vicinity. Among the current active applications in environment remediation and biomedicine, O_2_^•‒^ and/or ^1^O_2_ photogeneration from fluorescent proteins could highly be useful for inactivating harmful microorganisms and pathogens, such as bacteria, viruses, and fungi [111, 112], as well as contaminants and endocrine disrupting compounds [113]. In particular, ROS (i.e. ^1^O_2_) can be effective in inactivating viruses, impairing genome replication [114–117]. ROS could be useful for insect eradication [118, 119] and water disinfection for control of water-borne pathogens [120, 121].

Protein photosensitization can offer several pivotal advantages over conventional photocatalysis: (i) Fluorescent proteins can rule out biohazardous concerns on the byproducts and residuals of foreign synthesized metal/semiconductor nanomaterial photocatalysts. Thus, fluorescent proteins can overcome the limitation of hazardous and adverse (e.g. carcinogenic and cytotoxic) effects associated with photocatalytic nanoparticles [23, 24]. Fluorescent proteins are degradable and digestible, eliminating the potential risk of exposure and consumption. (ii) Without a need of additional nanoconjugations (e.g. mNPs, photosensitizers, and quantum dots), fluorescent proteins can generate selective ROS by being activated under solar (visible) light without UV irradiation. (iii) As ROS-generating nanomaterials, fluorescent proteins could potentially be mass-produced in an eco-friendly manner using biological reactors (e.g. microorganisms and insects) [71, 103, 122–124].
